# Comparison of DSM-IV and DSM-5 criteria for alcohol use disorders in VA primary care patients with frequent heavy drinking enrolled in a trial

**DOI:** 10.1186/s13722-017-0082-0

**Published:** 2017-07-18

**Authors:** Traci Takahashi, Gwen Lapham, Laura J. Chavez, Amy K. Lee, Emily C. Williams, Julie E. Richards, Diane Greenberg, Anna Rubinsky, Douglas Berger, Eric J. Hawkins, Joseph O. Merrill, Katharine A. Bradley

**Affiliations:** 1 Health Services Research and Development (HSR&D), Seattle Center of Innovation for Veteran-Centered and Value-Driven Care, 1660 S. Columbian Way, Seattle, WA 98108 USA; 2 Center of Excellence in Substance Abuse Treatment and Education (CESATE), Veterans Affairs (VA) Puget Sound Health Care System, S-123-PCC, 1660 S. Columbian Way, Seattle, WA 98108 USA; 3 Kaiser Permanente Washington Health Research Institute, 1730 Minor Avenue, Ste. 1600, Seattle, WA 98101 USA; 4 Department of Veterans Affairs Puget Sound Health Care System, General Medicine Services, 1660 South Columbian Way (S-152), Seattle, WA 98108 USA; 5 Department of Health Services, University of Washington, 1959 Pacific Street, Seattle, WA 98195 USA; 6 Department of Medicine, University of Washington, 1959 NE Pacific Street, Seattle, WA 98195 USA; 7 The Kidney Health Research Collaborative, San Francisco and San Francisco VA Medical Center, University of California, 4150 Clement Street (111A1), San Francisco, CA 94121 USA; 8 Psychiatry and Behavioral Sciences, University of Washington, Seattle, WA USA; 9 Department of Veterans Affairs Puget Sound Health Care System, Mental Health Service, 1660 South Columbian Way (S-152), Seattle, WA 98108 USA; 10 Division of Health Services Management and Policy, College of Public Health, The Ohio State University, Columbus, OH USA; 11 Center for Innovation in Pediatric Practice, Nationwide Children’s Hospital, Columbus, OH USA

**Keywords:** Alcohol use disorder, Binge drinking, DSM-IV, DSM-5, Alcohol

## Abstract

**Background:**

Criteria for alcohol use disorders (AUD) in the Diagnostic and Statistical Manual of Mental Disorders, 5th edition (DSM-5) were intended to result in a similar prevalence of AUD as DSM-IV. We evaluated the prevalence of AUD using DSM-5 and DSM-IV criteria, and compared characteristics of patients who met criteria for: neither DSM-5 nor DSM-IV AUD, DSM-5 alone, DSM-IV alone, or both, among Veterans Administration (VA) outpatients in the Considering Healthier drinking Options In primary CarE (CHOICE) trial.

**Methods:**

VA primary care patients who reported frequent heavy drinking and enrolled in the CHOICE trial were interviewed at baseline using the DSM-IV Mini International Neuropsychiatric Interview for AUD, as well as questions about socio-demographics, mental health, alcohol craving, and substance use. We compared characteristics across 4 mutually exclusive groups based on DSM-5 and DSM-IV criteria.

**Results:**

Of 304 participants, 13.8% met criteria for neither DSM-5 nor DSM-IV AUD; 12.8% met criteria for DSM-5 alone, and 73.0% met criteria for both DSM-IV and DSM-5. Only 1 patient (0.3%) met criteria for DSM-IV AUD alone. Patients meeting both DSM-5 and DSM-IV criteria had more negative drinking consequences, mental health symptoms and self-reported readiness to change compared with those meeting DSM-5 criteria alone or neither DSM-5 nor DSM-IV criteria.

**Conclusions:**

In this sample of primary care patients with frequent heavy drinking, DSM-5 identified 13% more patients with AUD than DSM-IV. This group had a lower mental health symptom burden and less self-reported readiness to change compared to those meeting criteria for both DSM-IV and DSM-5 AUD.

*Trial Registration* ClinicalTrials.gov NCT01400581. 2011 February 17

**Electronic supplementary material:**

The online version of this article (doi:10.1186/s13722-017-0082-0) contains supplementary material, which is available to authorized users.

## Background

**Fig. 1:**
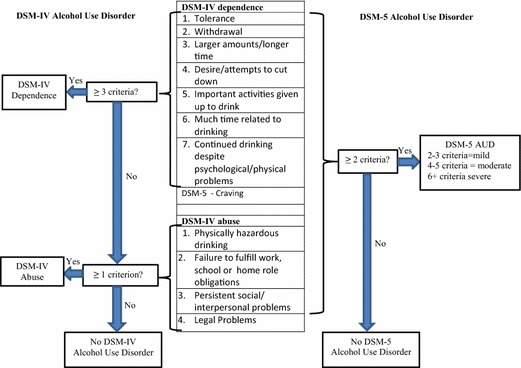
DSM-IV and DSM-5 alcohol use disorder criteria (AUD)

The Diagnostic and Statistical Manual of Mental Disorders, 5th edition (DSM-5) criteria for alcohol use disorders (AUD), released in 2013 [1], represent a significant departure from previous criteria. For the previous 20 years, since the 4th edition of the DSM (DSM-IV), alcohol dependence and abuse had been considered mutually exclusive diagnoses that together made up alcohol use disorders [2]. The diagnosis of these distinct disorders was based on “a maladaptive pattern of alcohol use leading to clinically significant impairment or distress” as manifested by separate criteria, with DSM-IV dependence requiring at least 3 of 7 criteria and DSM-IV alcohol abuse requiring exclusion of DSM-IV dependence and at least 1 of 4 separate criteria (Fig. 1). In part because of recent studies calling into question the hierarchical distinction between abuse and dependence [3–5], DSM-5 replaces these two diagnoses with a single spectrum of AUD with a continuum of severity. Removal of “abuse” from DSM-5 may also serve to reduce the stigma and negative judgment associated with such terminology [6]. The DSM-5 includes 11 criteria: 10 of the 11 combined DSM-IV abuse and dependence criteria (excluding legal problems) and a new criterion for craving (Fig. 1) [7]. DSM-5 further specifies AUD severity as mild, moderate or severe based on the number of diagnostic criteria endorsed, with at least 2 of 11 criteria required for a diagnosis (mild = 2–3, moderate = 4–5, severe ≥6). DSM-5 AUD was designed to reduce the number of “diagnostic orphans” that occurred with DSM-IV, whereby patients with 1 or 2 dependence criteria (and no abuse criteria) did not meet diagnostic criteria for abuse or dependence [8, 9]. Diagnostic orphans have an increased risk of developing a DSM-IV alcohol use disorder compared to those with no AUD symptoms in certain populations [e.g. young adults] and are therefore an important group to identify [10]. However, those who had DSM-IV abuse based on 1 criterion will not meet criteria for DSM-5 AUD [11]. The design of the DSM-5 AUD criteria is such that all people who met criteria for DSM-IV dependence but only some people with DSM-IV abuse will have an AUD based on DSM-5 criteria, while additional patients with only 2 symptoms of dependence will meet criteria.

DSM-5 criteria were intended to result in an overall prevalence of AUD similar to the prevalence of AUD determined from DSM-IV criteria [8]. A recent review found 12 studies that compared the prevalence of AUD based on DSM-IV and DSM-5 criteria [12]. Seven of the studies showed an increase in prevalence of AUD based on DSM-5 compared to DSM-IV, and the authors of the review concluded that DSM-5 “inflated” rates of AUD [12]. However, only one of the studies reviewed included a medical sample—patients seeking care in an emergency department. In contrast, the prevalence of AUD using DSM-5 criteria was found to be lower compared to DSM-IV criteria among high-risk Swiss young men [13] and in a large cross-national sample from the World Health Organization’s World Mental Health Survey Initiative [14]. None of these studies were restricted to primary care patients and clinical samples could differ (e.g. have more severe AUD) which could decrease the impact of the shift from DSM-IV to DSM-5.

Even if a difference in the prevalence of DSM-IV and DSM-5 AUD is not observed in other settings, patients identified by the two diagnostic criteria could have different demographic or clinical profiles. Little research has focused on demographic and clinical differences between patients who meet DSM-IV and DSM-5 criteria for AUD in clinical settings. Both the change in number of diagnostic criteria required, and the change from “legal problems” to “craving,” could result in differences in the clinical characteristics of the populations of patients diagnosed with DSM-5 AUD and DSM-IV AUD.

The purpose of the present study was to compare the characteristics of patients diagnosed with AUD according to DSM-IV and DSM-5 criteria in a clinical sample of US Veterans who received general medical care (e.g. “primary care”) in Veterans Affairs (VA) clinics and reported frequent heavy drinking and were recruited into a trial of alcohol-related care management. We first describe the prevalence of DSM-IV and DSM-5 AUD, and categorize patients into four mutually exclusive groups: those who met criteria for: (1) neither DSM-IV nor DSM-5 AUD; (2) DSM-IV AUD alone; (3) DSM-5 AUD alone; or (4) both DSM-IV and DSM-5 AUD. We then compare sociodemographic characteristics, alcohol and drug related problem severity, mental health comorbidity, and readiness to change drinking across the four mutually exclusive diagnostic groups.

## Methods

The Considering Healthier Drinking Options in primary CarE (aka “CHOICE”) trial was a randomized controlled trial of a 12 months nurse-led care management intervention for patients with frequent heavy drinking who were receiving medical care in a general practice clinic for US Veterans of military service. Analyses presented here are from the baseline interviews (December 2011–September 2014) that assessed both DSM-IV and DSM-5 alcohol use disorders. The study was approved by both the VA Puget Sound and the Group Health Institutional Review Boards.

### Study sample and setting

The CHOICE trial was conducted at three VA primary care clinics (Seattle, Tacoma, and Mount Vernon, Washington). Patients were considered potentially eligible if they were 21–75 years old and had a positive alcohol screen documented at the time of a VA outpatient visit (Alcohol Use Disorders Identification Test Consumption questionnaire [AUDIT-C] score ≥4 points for women and ≥5 for men). These cut-offs were chosen to maximize the positive predictive value for AUD. [15] In addition, providers could refer patients and flyers were posted so patients could self-refer to the study. Potentially eligible and referred patients were contacted by telephone and determined to be eligible if they reported drinking at levels that exceeded National Institute on Alcohol Abuse and Alcoholism (NIAAA) daily drinking limits on a phone screen—4 drinks (women) or 5 drinks (men), hereafter “binge”—at least twice a week on average, or once a week on average if they also reported they had ever been in alcohol treatment or attended Alcoholics Anonymous [16]. This was used as a proxy screen for AUD for the CHOICE trial because, due to stigma [17], AUD are long known to be under-diagnosed clinically [18]. Patients were excluded if—based on either chart review or telephone screening—they had cognitive impairment, did not speak English, were acutely unstable medically or psychiatrically, had less than 1 year life expectancy, were a VA employee, were enrolled in another VA intervention study, had been in alcohol treatment in the previous 90 days (including medications for AUD, but not Alcoholics Anonymous or 12 step meetings), were planning on becoming pregnant or currently pregnant, had no contact with their primary care team in the last year, or were not planning to continue care at VA Puget Sound facilities in the next year. Eligible patients provided written informed consent at a baseline enrollment visit, followed by repeat assessment of eligibility and self- and interviewer-administered questionnaires.

## Measures

### DSM-IV and DSM-5 alcohol use disorders (AUD) diagnoses

The Mini-International Neuropsychiatric Interview (MINI), a brief interviewer-administered diagnostic interview, was used to assess past-year AUD. The DSM-IV MINI includes 7 questions assessing dependence criteria in the past 12 months and then—if patients report 0–2 symptoms and therefore do not meet criteria for DSM-IV dependence—they are asked 4 questions to assess DSM-IV alcohol abuse criteria. This skip pattern minimizes questionnaire burden.

Although the final DSM-5 criteria for AUD were not published at the time the CHOICE trial began, the addition of a craving criterion was expected. All CHOICE trial participants were therefore also asked the following question about craving, after the DSM-IV MINI: “Was there ever a time in your life when you often had such a strong desire to drink that you couldn’t stop yourself from taking a drink or found it difficult to think of anything else?” from the National Epidemiologic Survey on Alcohol and Related Conditions (NESARC) [19]. Of note, the question’s timeframe of “ever” was inadvertently not changed to the “past year” for use in our measure of past year DSM-5 AUD, and the prevalence of lifetime craving is likely greater than past year craving.

Patients were categorized as having past year DSM-IV AUD using standard DSM-IV criteria: 3 or more criteria for alcohol dependence or 1 or more criteria for alcohol abuse for those who did not meet alcohol dependence criteria. Patients were categorized as having a DSM-5 AUD if they met 2 or more of the 11 DSM-5 criteria (Fig. 1). The severity of DSM-5 AUD is not reported because it may be under-estimated in patients who met DSM-IV dependence criteria, because they were not asked the 3 DSM-IV abuse questions included in the 11 DSM-5 criteria for AUD.

### Socio-demographics, smoking, mental health symptoms and drug use disorders

Interviewers assessed demographic characteristics including gender, race, marital status, education and income, as well as whether patients smoked tobacco every day, some days or not at all. Patients also completed the following self-administered screening questionnaires: the 9-item Patient Health Questionnaire (PHQ-9; ≥10 points positive screen for depression), the 7-item Generalized Anxiety Disorder Screen (GAD-7; ≥10 points positive screen), and the Post-Traumatic Stress Disorder (PTSD) Checklist (PCL-C; ≥50 points positive screen). Interviewers also administered the panic disorder and drug use disorders (DUD) modules of the DSM-IV MINI. A count measure of the number of mental health and drug use conditions was constructed by summing the total number of positive screens or diagnoses (depression, generalized anxiety, PTSD, panic, and DUD).

### Negative alcohol-related consequences, treatment history, and readiness to change

The Short Inventory of Problems (SIP) was used to assess each of 15 negative consequences due to drinking in the past 3 months and ever in the patient’s lifetime [20–23]. The number of adverse alcohol-related consequences were summed as a measure of the total number of reported negative consequences in the past 3 months and ever. We used this descriptive measure because it reflected the number of symptoms, rather than the total SIP score which is more abstract because it takes into account both the number of negative consequences and severity. Interviewers also assessed prior treatment utilization by asking: “Have you ever gone anywhere or seen anyone for a reason that was related in any way to your drinking: a physician, counselor, Alcoholics Anonymous, or any other community agency or professional?” with 3 response options: No; Yes, prior to the past year; and Yes, during the past year [24]. Three 10-point Likert scale readiness “rulers,” [25, 26] adapted from a previous primary care trial [27], were used to assess readiness to change, the importance of change, and confidence in the ability to change. For example, response options for the question “How important is it to you right now to change your drinking?” included 0 = “I don’t drink; does not apply;” 1–3 = “not important;” 4–6 = “somewhat important;” and 7–10 = “very important.” For this analysis, responses were dichotomized as: somewhat or ready to change (4–10) versus not (1–3); somewhat or very important to change (4–10) versus not (1–3); and somewhat or very confident in ability to change (4–10) versus not (1–3).

### Analyses

We describe the socio-demographic and clinical characteristics of patients who enrolled in the CHOICE study and the prevalence of DSM-IV and DSM-5 AUD. We then categorize patients into 4 mutually exclusive groups: (1) those meeting neither DSM-IV nor DSM-5 criteria for AUD; (2) those meeting criteria for DSM-IV AUD alone; (3) those meeting criteria for DSM-5 AUD alone; and (4) those meeting criteria for both DSM-IV and DSM-5 AUD.

Finally, we describe the socio-demographic and clinical characteristics, including smoking, mental health and drug use comorbidity, negative consequences due to drinking and alcohol treatment history, across the 4 groups. The potential impact of asking about lifetime craving (instead of past year) was addressed in sensitivity analyses that omitted the craving question and used only 10 criteria for DSM-5 AUD. These analyses used the same threshold of two or more criteria to diagnose DSM-5 AUD.

In order to evaluate differences in characteristics across the mutually exclusive diagnostic groups—neither DSM-IV nor DSM-5 AUD, DSM-5 AUD alone, and both DSM-IV and DSM-5 AUD—three post hoc statistical tests were conducted using 3 aggregate outcome measures to avoid multiple statistical comparisons. The three outcomes for these post hoc analyses were: 1) number of mental health or drug use conditions (out of 5 possible); 2) number of negative alcohol-related consequences in the past-3 months on the SIP (out of 15 possible); and 3) an indicator of whether or not patients reported feeling “somewhat” to “ready” to change (versus not ready). Three generalized linear models were constructed, one for each of the 3 aggregate outcomes, with the categorical variable for the remaining DSM-IV and/or DSM-5 AUD groups as the independent variable, using the group with neither DSM-IV nor DSM-5 AUD as the referent. The outcomes (number of events; binary indicator) were assumed to have a binomial distribution, with the denominator equal to the number of possible events, and a logistic link was used. An overall post-estimation Wald test then evaluated whether the categorical variable for the mutually exclusive diagnostic groups was significant (defined as p < 0.05). With the exception of four missing responses for the PTSD Checklist, there were no other missing data.

## Results

### Characteristics of study sample of patients

Most of the 304 study participants were male (90.5%) and a majority (60.9%) were at least 50 years old (Table 1). Almost one-half (45.4%) screened positive for depression (PHQ-9 ≥ 10), nearly one-third (30.3%) screened positive for generalized anxiety (GAD ≥ 10) and the same proportion (30.3%) screened positive for PTSD (PCL ≥ 50). About one in 10 (9.5%) had symptoms consistent with DSM-IV panic disorder, while nearly one-fifth (18.8%) met criteria for a past year DSM-IV drug use disorder.

**Table 1:** Characteristics of study population: VA primary care patients enrolled in the CHOICE trial (n = 304)

	n	(%)
Female	29	(9.5)
Age categories		
21–34	54	(17.8)
35–49	65	(21.4)
50–64	131	(43.1)
65+	54	(17.8)
Patient-reported race		
Native American	25	(8.2)
Asian	2	(0.7)
Native Hawaiian/Pacific Islander	5	(1.6)
Black	39	(12.8)
White	206	(67.8)
Multiracial	22	(7.2)
Other	5	(1.6)
Hispanic/Latino	21	(6.9)
Marital status		
Never married	56	(18.4)
Married/partnered	136	(44.7)
Separated	13	(4.3)
Divorced	91	(29.9)
Widowed	7	(2.3)
Refused/unknown	1	(0.3)
Education		
High school/GED or less	65	(21.4)
Some college/tech school	170	(55.9)
College or post graduate	69	(22.7)
Income		
<$15,000	49	(16.1)
$15,000-59,999	158	(52.0)
≥$60,000	95	(31.3)
Refused/Unknown	2	(0.7)
Smokes tobacco currently	134	(44.1)
Depression screen positive (PHQ-9 ≥ 10)	138	(45.4)
Generalized anxiety screen positive (GAD-7 ≥ 10)	92	(30.3)
PTSD screen positive (PCL-C ≥ 50)^a^	91	(30.3)
DSM-IV panic disorder– current (MINI)	29	(9.5)
DSM-IV drug use disorders past year (MINI)	57	(18.8)

^a^Four patients did not complete the PCL

### Prevalence of DSM-IV and DSM-5 alcohol use disorders

Overall, 85.9% of patients met criteria for DSM-5 AUD, whereas 73.3% met criteria for DSM-IV AUD. Of those with DSM-IV AUD, 81% met criteria for dependence and 19% for abuse alone. Table 2 shows the number of patients in each of the 4 mutually exclusive diagnostic groups: 13.8% met neither DSM-IV nor DSM-5 criteria for AUD; 0.3% met criteria for DSM-IV AUD alone, 12.8% met criteria for DSM-5 AUD alone, and 73.0% met criteria for both DSM-IV and DSM-5 AUD. The lone patient meeting criteria for DSM-IV AUD alone endorsed alcohol use despite social/interpersonal problems (1 of the 4 DSM-IV abuse criteria). Of note, in sensitivity analyses, 32 of 39 (82%) patients who met criteria for DSM-5 AUD alone still met DSM-5 criteria using only 10 DSM-5 criteria (craving question omitted because it asked about a time frame “ever” rather than past year).

**Table 2:** Comparison of DSM-IV and DSM-5 alcohol use disorder

	DSM-5 diagnosis					
	No		Yes		Total	
	n	(%)	n	(%)	n	(%)
DSM-IV diagnosis						
No	42	(97.7)	39	(14.9)	81	(26.6)
Yes	1	(2.3)	222	(86.1)	223	(73.3)
Total	43	(14.1)^a^	261	(85.9)^a^	304	(100.0)

^a^Designates row percentages (the remainder of percentages in this table are column percentages)

### Comparison of the 4 groups based on DSM-IV and DSM-5 criteria for AUD

#### Sociodemographic Characteristics

Comparison of sociodemographic characteristics across the 4 diagnostic groups—neither DSM-IV nor DSM-5 AUD, DSM-IV AUD alone, DSM-5 AUD alone, or both—suggested differences in age, race, marital status, and income (Table 3a). Because only 1 patient met criteria for DSM-IV alone, we compare the other 3 groups in the description of the remainder of results. Compared to patients with both DSM-IV and DSM-5 AUD, patients meeting neither DSM-IV nor DSM-5 AUD were more likely to be age 65 or more, and more likely to report white race, being married, and higher incomes (Table 3a).

**Table 3:** Sociodemographic, Smoking, Mental Health and Substance Use Characteristics of Patients Meeting Criteria for Neither DSM-IV nor DSM-5 AUD, DSM-IV AUD alone, DSM-5 AUD alone, or Both

	Neither DSM-IV nor DSM-5 n = 42		DSM-IV AUD alone n = 1		DSM-5 AUD alone n = 39		Both DSM-IV & DSM-5 AUD n = 222	
(*a*)								
Female	5	(11.9)	0	(0.0)	4	(10.3)	20	(9.0)
Age categories								
21–34	4	(9.5)	0	(0.0)	5	(12.8)	45	(20.3)
35–49	7	(16.7)	1	(100.0)	10	(25.6)	47	(21.2)
50–64	13	(31.0)	0	(0.0)	13	(33.3)	105	(47.3)
65+	18	(42.9)	0	(0.0)	11	(28.2)	25	(11.3)
Patient-reported race								
Native American	1	(2.4)	0	(0.0)	5	(12.8)	19	(8.6)
Asian	0	(0.0)	0	(0.0)	0	(0.0)	2	(0.9)
Native Hawaiian/Pacific Islander	1	(2.4)	0	(0.0)	0	(0.0)	4	(1.8)
Black	3	(7.1)	1	(100.0)	6	(15.4)	29	(13.1)
White	36	(85.7)	0	(0.0)	28	(71.8)	142	(64.0)
Multiracial	1	(2.4)	0	(0.0)	0	(0.0)	21	(9.5)
Other	0	(0.0)	0	(0.0)	0	(0.0)	5	(2.3)
Hispanic/Latino	3	(7.1)	0	(0.0)	2	(5.1)	16	(7.2)
Marital status								
Never married	7	(16.7)	0	(0.0)	8	(20.5)	41	(18.5)
Married/partnered	22	(52.4)	0	(0.0)	18	(46.2)	96	(43.2)
Separated	1	(2.4)	0	(0.0)	2	(5.1)	10	(4.5)
Divorced	10	(23.8)	1	(100.0)	9	(23.1)	71	(32.0)
Widowed	2	(4.8)	0	(0.0)	2	(5.1)	3	(1.4)
Refused/unknown	0	(0.0)	0	(0.0)	0	(0.0)	1	(0.5)
Education								
High school/GED or less	9	(21.4)	0	(0.0)	9	(23.1)	47	(21.2)
Some college/tech school	20	(47.6)	1	(100.0)	20	(51.3)	129	(58.1)
College or post graduate	13	(31.0)	0	(0.0)	10	(25.6)	46	(20.7)
Income								
<$15,000	3	(7.1)	1	(100.0)	5	(12.8)	40	(18.0)
$15,000–59,999	20	(47.7)	0	(0.0)	22	(56.4)	116	(52.3)
≥$60,000	18	(42.9)	0	(0.0)	12	(30.8)	65	(29.3)
Refused/unknown	1	(2.4)	0	(0.0)	0	(0.0)	1	(0.5)
(*b*)								
Smokes currently	11	(26.2)	1	(100.0)	15	(38.5)	107	(48.2)
Depression symptoms (PHQ-9 ≥ 10)	3	(7.1)	0	(0.0)	5	(12.8)	130	(58.6)
Generalized anxiety symptoms (GAD-7 ≥ 10)	4	(9.5)	0	(0.0)	0	(0.0)	88	(39.7)
DSM-IV panic disorder—current (MINI)	1	(2.4)	0	(0.0)	6	(15.4)	84	(38.4)
DSM-IV drug use disorders past year (MINI)	2	(4.8)	0	(0.0)	1	(2.6)	26	(11.7)
Count of mental health and drug use conditions*, mean (SD)**	0	(0.0)	0	(0.0)	2	(5.1)	55	(24.8)
Negative alcohol-related consequences*	0.2	(0.1)	0	(0.0)	0.4	(0.1)	1.7	(0.1)
Negative alcohol-related consequences								
Short Inventory of Problems (SIP)—past 3 months, mean (SD)**	1.0	(0.3)	1	n/a	2.5	(0.4)	6.8	(0.3)
Short inventory of problems (SIP)—lifetime, mean (SD)	4.2	(0.5)	5	n/a	6.7	(0.5)	10.1	(0.3)
Never seen anyone/gone anywhere for drinking-related reason?	33	(78.6)	0	(0.0)	21	(53.8)	81	(36.5)
Somewhat or ready to change**	17	(40.5)	0	(0.0)	24	(61.5)	165	(74.3)
Somewhat or very Important to change	15	(35.7)	0	(0.0)	24	(61.5)	178	(80.2)
Somewhat or very confident in ability to change	39	(92.9)	1	(100.0)	35	(89.7)	171	(77.0)

* Sum of the total number of positive screens for depression, generalized anxiety, PTSD, panic, and DUD

** Wald test p < 0.0

#### Smoking, mental health symptoms and drug use disorders

Those with DSM-5 AUD alone appeared to have rates of smoking, mental health symptoms, and drug use disorders that tended to be higher than those who met criteria for neither DSM-IV nor DSM-5 AUD but lower than those who met criteria for both DSM-IV and DSM-5 (Table 3b).

#### Alcohol-related negative consequences

Table 3b shows the mean frequency for each consequence due to drinking in the past 3 months as reported on the SIP. For patients who met criteria for neither DSM-IV nor DSM-5 AUD, DSM-5 alone, and both DSM-IV and DSM-5 AUD, the mean number of alcohol-related negative consequences in the past 3 months was 1.0 (SD 0.3), 2.5 (SD 0.4), and 6.8 (SD 0.3) respectively. The mean number of lifetime negative consequences due to drinking for patients who met criteria for neither DSM-IV nor DSM-5 AUD, DSM-5 alone, and both DSM-IV and DSM-5 AUD, was 4.2 (SD 0.5), 6.7 (SD 0.5), and 10.1 (SD 0.3) respectively. The prevalence of having never sought help due to drinking among those with neither DSM-IV nor DSM-5 AUD, DSM-5 AUD alone, and both DSM-IV and DSM-5 AUD, was 78.6%, 53.8% and 36.5%, respectively.

#### Readiness to change and confidence in ability to change

As shown in Table 3b, the readiness to change drinking for patients who met criteria for neither DSM-IV nor DSM-5 AUD, DSM-5 alone, and both DSM-IV and DSM-5 AUD, was 40.5%, 61.5% and 74.3% respectively. While 80.2% of patients who met criteria for both DSM-IV and DSM-5 AUD reported that change was somewhat or very important, 61.5% of those with DSM-5 AUD alone and 35.7% of those who met neither DSM-IV nor DSM-5 criteria for AUD reported change was somewhat or very important. However, patients who met both DSM-IV and DSM-5 criteria were least likely to report being somewhat or very confident in their ability to change (77.0%), while those who met DSM-5 criteria alone and those who met neither DSM-IV nor DSM-5 criteria for AUD appeared to have similar rates of reporting being somewhat or very confident in their ability to change (92.9 and 89.7% respectively).

#### Post-hoc analyses

Because descriptive analyses suggested that patients who met criteria for DSM-5 alone may differ on the burden of negative consequences due to drinking, mental health and drug use conditions, and readiness to change from patients meeting criteria for neither DSM-IV nor DSM-5 AUD, or both DSM-IV and DSM-5 AUD, we performed post hoc statistical tests to evaluate whether differences across the mutually exclusive diagnostic groups were statistically significant. For the purposes of these post hoc analyses, the diagnostic group that included the lone subject who met DSM-IV criteria for AUD alone, was excluded resulting in a 3-way comparison. These analyses revealed that all three measures differed significantly across the 3 groups (p < 0.001) (Table 3b).

## Discussion

In this sample of VA primary care patients at high risk for AUD due to frequent binge drinking, DSM-5 AUD criteria identified about 13% more patients with AUD than DSM-IV criteria. Almost three quarters of patients met both DSM-IV and DSM-5 criteria for AUD and 14% met criteria for neither, but only one patient out of 304 met criteria for DSM-IV AUD but not DSM-5 AUD. Moreover, in this sample of primary care patients recruited for a trial of care management for patients at high risk for AUD, there were marked differences in the clinical characteristics across the four groups. Those who met both DSM-IV and DSM-5 AUD criteria not only had more negative consequences due to drinking, but also more mental health and drug use symptoms and greater reported readiness to change compared with those who met either DSM-IV or DSM-5 AUD alone, and those who met neither DSM-IV nor DSM-5 criteria for AUD.

A unique strength of this study is that it compared DSM-IV and DSM-5 criteria in a clinical sample of heavy drinkers. This is an important population to study because it is these patients in whom assessments for AUD are used clinically. It is not possible to determine the extent to which the higher prevalence of AUD using DSM-5 criteria (85.9%) compared to DSM-IV criteria (73.3%) is due to the specific characteristics of the sample we studied—primary care patients who reported frequent binge drinking—or if this result would be found in more general clinical populations. However, this study’s finding that a sizable group of patients (13%) met criteria for DSM-5 AUD alone is consistent with 7 of 12 recent studies comparing the prevalence of AUD based on DSM-IV and DSM-5 criteria in mostly non-clinical settings [12]. The fact that only one patient met criteria for DSM-IV AUD alone might be somewhat surprising. One might have expected more than 1 patient with frequent binge drinking to meet the DSM-IV AUD criteria with just 1 DSM-IV abuse criterion or 2 DSM-IV criteria that included legal consequences, which was excluded from DSM-5. However, drinking 5 or more drinks on an occasion is strongly associated with AUD symptoms [3], and thus restriction of our sample to frequent binge drinking could account for this finding. Further research is needed on other clinical samples of patients with frequent binge drinking to understand if this unexpected finding was unique to our study sample.

Patients meeting criteria for both DSM-IV and DSM-5 AUD had relatively high rates of mental health symptoms, drug use disorders, and recent negative consequences due to drinking. Moreover, almost three quarters of these patients who met both DSM-IV and DSM-5 criteria were somewhat or very interested in changing their drinking. In contrast, patients who met criteria for DSM-5 AUD alone, who by definition met criteria for mild DSM-5 AUD, tended to report fewer negative consequences due to drinking, less tobacco use and drug use disorders, and fewer symptoms of anxiety and depression. This is an expected finding since DSM-5 was designed to better capture less severe forms of AUD and to have dimensionality such that participants with fewer DSM criteria have a milder AUD [8–10]. Further, patients who met criteria for DSM-5 AUD alone had less readiness to change compared to patients who met both DSM-IV and DSM-5 criteria, despite having similar demographic characteristics. This association between alcohol-related symptom burden and readiness to change has been previously described [28], but the finding that DSM-5 identifies more of these patients with milder alcohol-related symptoms who report less readiness to change may have clinical implications for primary care. Given the lower burden of mental health symptoms reported by patients with DSM-5 AUD alone, and their greater confidence in their ability to change their drinking, patients meeting DSM-5 criteria alone may be more likely to respond to brief opportunistic interventions in primary care compared to those who meet criteria for both DSM-IV and DSM-5 AUD. On the other hand, these patients tended to report less treatment-seeking and placing less importance on changing their drinking than those meeting both DSM-IV and DSM-5 AUD criteria and they were less likely to report that they were ready to change. These characteristics may make patients meeting criteria for DSM-5 alone more successful at self-change, but more challenging to engage in opportunistic primary care interventions—like the CHOICE trial intervention–which seek to engage patients repeatedly over time and/or interest them in medications for AUD [29, 30]. Future research will be needed to assess whether the effectiveness of the CHOICE intervention differed for those who met criteria for DSM-5 AUD alone vs both DSM-IV and DSM-5 AUD.

In this sample of primary care patients who reported frequent binge drinking and enrolled in a trial, 14% did not meet criteria for DSM-IV or DSM-5 AUD. To our knowledge, little research has focused on this group. These patients had similar rates of mental health co-morbidity compared to those with an AUD based on DSM-5 criteria alone. However, they tended to be older and reported a mean of 4 of 15 negative consequences due to drinking in their lifetimes, suggesting that they may have had prior AUD now in remission. Patients with frequent binge drinking who do not meet DSM criteria for AUD *on average* respond to opportunistic brief interventions [31], but little is known about whether older patients who have previously experienced negative consequences due to drinking and may have AUD in remission respond to brief interventions. Future research is also needed to evaluate whether alcohol medications can decrease drinking and future negative consequences related to drinking for this group.

This study has several important limitations. First, the study sample reflects VA primary care patients with frequent binge drinking who were willing to enroll in a trial in which they would be offered alcohol-related services, limiting generalizability. The prevalence of AUD based on DSM-IV and DSM-5 is likely to be markedly lower in general primary care populations, and may differ in younger and non-research populations. It is also possible that those patients meeting criteria for DSM-IV AUD alone (1 DSM-IV abuse criterion or 2 DSM-IV criteria including legal consequences) were less likely to participate in a research study on alcohol and therefore were not represented in our sample. For example, this could disproportionately impact racial minorities who may be more likely to have legal consequences of drinking [32], but less likely to participate in trials. The recruited sample was also 90% men, who tend have higher rates of AUD than women, and the sample had a relatively high prevalence of mental health symptoms and drug use disorders.

Several limitations of this study’s measures should also be noted. The DSM-IV skip pattern limited our ability to measure DSM-5 AUD severity in those with DSM-IV alcohol dependence, because the interview skip pattern that did not assess the 4 questions about abuse in patients with dependence. In addition, we used a craving question with a lifetime timeframe. However, sensitivity analyses determined that the impact of the “ever” time frame of the craving question had minimal impact on our findings (Additional file 1, Additional file 2). This descriptive study also lacked a priori hypotheses about differences across the four mutually exclusive groups and did not have power to test multiple comparisons. Additional studies of the prevalence and severity of DSM-5 AUD and associated mental health comorbidity and readiness to change in primary care patients with frequent binge drinking will be important (Additional file 3).

## Conclusions

Despite the above limitations, these findings may be pertinent to patients with frequent binge drinking in the VA, the largest integrated health system in the US with over 900 clinical sites nationwide. As above, this is also the first study to compare DSM-IV and DSM-5 criteria in a primary care population to our knowledge. Additionally, primary care patients with frequent binge drinking are an important clinical sample in which to study DSM diagnostic criteria. Most brief alcohol screens assess frequent or recent heavy drinking and a follow-up diagnostic assessment for AUD can aid treatment decisions (i.e. brief intervention vs. medication) [33]. Further, results of this study highlight the spectrum and complexity of patients with frequent binge drinking who might be open to receiving alcohol-related care in their primary care clinic.

In sum, in this study of VA primary care patients with frequent binge drinking, 13% of patients met DSM-5 AUD criteria but did not meet DSM-IV AUD criteria. These patients appeared to have fewer negative consequences due to drinking and less other substance use and mental health comorbidity, than patients who met criteria for both DSM-IV and DSM-5 AUD. If findings are corroborated by future primary care studies, developing and testing optimal approaches to managing these primary care patients with less complex AUD may be important.

## Additional files

**Additional file 1.** Sociodemographic Characteristics of Patients Meeting Criteria for Neither DSM-IV nor DSM-5 AUD, DSM-IV AUD alone, DSM-5 AUD alone, or Both when the craving question (with a timeframe of “ever”) is omitted from DSM-5.

**Additional file 2.** Smoking, Mental Health and Substance Use Characteristics of Patients Meeting Criteria for Neither DSM-IV nor DSM-5 AUD, DSM-IV AUD alone, DSM-5 AUD alone, or Both when the craving question (with a timeframe of “ever”) is omitted from DSM-5.

**Additional file 3.** Individual Symptoms from the Short Inventory of Problems for Patients Meeting Criteria for Neither DSM-IV nor DSM-5 AUD, DSM-IV AUD alone, DSM-5 AUD alone, or Both.

## Abbreviations

AUD: alcohol use disorders
DSM-IV: the diagnostic and statistical manual of mental disorders, 4th edition
DSM-5: the diagnostic and statistical manual of mental disorders, 5th edition
VA: veterans affairs
CHOICE: considering healthier drinking options in primary CarE
AUDIT-C: alcohol use disorders identification test consumption questionnaire
NESARC: national epidemiologic survey on alcohol and related conditions
NIAAA: national institute on alcohol abuse and alcoholism
MINI: mini-international neuropsychiatric interview
PHQ-9: 9-item patient health questionnaire
GAD: generalized anxiety disorder screen
PCL-C: post-traumatic stress disorder checklist
PTSD: post-traumatic stress disorder
DUD: drug use disorder
SIP: short inventory of problems
SD: standard deviation
