# Supplementary material for: Comparison of DSM-IV and DSM-5 criteria for alcohol use disorders in VA primary care patients with frequent heavy drinking enrolled in a trial
Source: Addict Sci Clin Pract. 2017 Jul 18;12:17. doi: 10.1186/s13722-017-0082-0 (PMC5514480; doi:10.1186/s13722-017-0082-0)
Supplement: Supplementary file 1 — Additional file 1. Sociodemographic Characteristics of Patients Meeting Criteria for Neither DSM-IV nor DSM-5 AUD, DSM-IV AUD alone, DSM-5 AUD alone, or Both when the craving question (with a timeframe of “ever”) is omitted from DSM-5. [file 13722_2017_82_MOESM1_ESM.docx]

Additional File 1. Sociodemographic Characteristics of Patients Meeting Criteria for Neither DSM-IV nor DSM-5 AUD, DSM IV AUD alone, DSM 5 AUD alone, or Both when the craving question (with a timeframe of “ever”) is omitted from DSM-5.

|  | | Neither DSM-IV nor DSM-5  n=49 | | DSM-IV  AUD alone  n=1 | | DSM-5  AUD alone  n=32 | | | Both DSM-IV & DSM-5 AUD  n=222 | | |
| --- | --- | --- | --- | --- | --- | --- | --- | --- | --- | --- | --- |
| Female | 6 | (12.2) | 0 | (0.0) | | 3 | (9.4) | | 20 | (9.0) |  |
| Age categories |  |  |  |  | |  |  | |  |  |  |
| 21-34 | 5 | (10.2) | 0 | (0.0) | | 4 | (12.5) | | 45 | (20.3) |  |
| 35-49 | 8 | (16.3) | 1 | (100.0) | | 9 | (28.1) | | 47 | (21.2) |  |
| 50-64 | 15 | (30.6) | 0 | (0.0) | | 11 | (34.4) | | 105 | (47.3) |  |
| 65+ | 21 | (42.9) | 0 | (0.0) | | 8 | (25.0) | | 25 | (11.3) |  |
| Patient-reported race |  |  |  |  | |  |  | |  |  |  |
| Native American | 2 | (4.1) | 0 | (0.0) | | 4 | (12.5) | | 19 | (8.6) |  |
| Asian | 0 | (0.0) | 0 | (0.0) | | 0 | (0.0) | | 2 | (0.9) |  |
| Native Hawaiian/Pacific Islander | 1 | (2.0) | 0 | (0.0) | | 0 | (0.0) | | 4 | (1.8) |  |
| Black | 5 | (10.2) | 1 | (100.0) | | 4 | (12.5) | | 29 | (13.1) |  |
| White | 40 | (81.6) | 0 | (0.0) | | 24 | (75.0) | | 142 | (64.0) |  |
| Multiracial | 1 | (2.0) | 0 | (0.0) | | 0 | (0.0) | | 21 | (9.5) |  |
| Other | 0 | (0.0) | 0 | (0.0) | | 0 | (0.0) | | 5 | (2.3) |  |
| Hispanic | 3 | (6.1) | 0 | (0.0) | | 2 | (6.3) | | 16 | (7.2) |  |
| Marital Status |  |  |  |  | |  |  | |  |  |  |
| Never Married | 7 | (14.3) | 0 | (0.0) | | 8 | (25.0) | | 41 | (18.5) |  |
| Married/Partnered | 25 | (51.0) | 0 | (0.0) | | 15 | (46.9) | | 96 | (43.2) |  |
| Separated | 1 | (2.0) | 0 | (0.0) | | 2 | (6.3) | | 10 | (4.5) |  |
| Divorced | 13 | (26.5) | 1 | (100.0) | | 6 | (18.8) | | 71 | (32.0) |  |
| Widowed | 3 | (6.1) | 0 | (0.0) | | 1 | (3.1) | | 3 | (1.4) |  |
| Refused/Unknown | 0 | (0.0) | 0 | (0.0) | | 0 | (0.0) | | 1 | (0.5) |  |
| Education |  |  |  |  | |  |  | |  |  |  |
| High school/GED or less | 9 | (18.4) | 0 | (0.0) | | 9 | (28.1) | | 47 | (21.2) |  |
| Some college/tech school | 26 | (53.1) | 1 | (100.0) | | 14 | (43.8) | | 129 | (58.1) |  |
| College or post graduate | 14 | (28.6) | 0 | (0.0) | | 9 | (28.1) | | 46 | (20.7) |  |
| Income |  |  |  |  | |  |  | |  |  |  |
| <$15,000 | 4 | (8.2) | 1 | (100.0) | | 4 | (12.5) | | 40 | (18.0) |  |
| $15,000-59,999 | 23 | (46.9) | 0 | (0.0) | | 19 | (59.4) | | 116 | (52.3) |  |
| >=$60,000 | 21 | (42.9) | 0 | (0.0) | | 9 | (28.1) | | 65 | (29.3) |  |
| Refused/Unknown | 1 | (2.0) | 0 | (0.0) | | 0 | (0.0) | | 1 | (0.0) |  |
