# Supplementary material for: Comparison of DSM-IV and DSM-5 criteria for alcohol use disorders in VA primary care patients with frequent heavy drinking enrolled in a trial
Source: Addict Sci Clin Pract. 2017 Jul 18;12:17. doi: 10.1186/s13722-017-0082-0 (PMC5514480; doi:10.1186/s13722-017-0082-0)
Supplement: Supplementary file 2 — Additional file 2. Smoking, Mental Health and Substance Use Characteristics of Patients Meeting Criteria for Neither DSM-IV nor DSM-5 AUD, DSM-IV AUD alone, DSM-5 AUD alone, or Both when the craving question (with a timeframe of “ever”) is omitted from DSM-5. [file 13722_2017_82_MOESM2_ESM.docx]

Additional File 2. Smoking, Mental Health and Substance Use Characteristics of Patients Meeting Criteria for Neither DSM-IV nor DSM-5 AUD, DSM IV AUD alone, DSM 5 AUD alone, or Both when the craving question (with a timeframe of “ever”) is omitted from DSM-5.

|  | Neither DSM-IV nor DSM-5  n=49 | | | DSM-IV  AUD alone  n=1 | | DSM-5  AUD alone  n=32 | | Both DSM-IV & DSM-5 AUD  n=222 | | | |
| --- | --- | --- | --- | --- | --- | --- | --- | --- | --- | --- | --- |
|  |  | |  |  |  |  |  |  | |  | |
| Smokes currently (n, %) | 11 | | (22.4) | 1 | (100.0) | 15 | (46.9) | 107 | | (48.2) | |
| Depression screen positive (PHQ-9 ≥ 10) | 3 | | (6.1) | 0 | (0.0) | 5 | (15.6) | 130 | | (58.6) | |
| Generalized anxiety screen positive (GAD-7 ≥ 10) | 4 | | (8.2) | 0 | (0.0) | 0 | (0.0) | 88 | | (39.6) | |
| PTSD screen positive (PCL-C ≥ 50) | 1 | | (2.1) | 0 | (0.0) | 6 | (18.8) | 84 | | (38.4) | |
| DSM-IV panic disorder – current (MINI) | 2 | | (4.1) | 0 | (0.0) | 1 | (3.1) | 26 | | (11.7) | |
| DSM-IV drug use disorders past year (MINI) | 0 | | (0.0) | 0 | (0.0) | 2 | (6.3) | 55 | | (24.8) | |
| Count of mental health and drug use conditions, mean (SD)* |  |  | | 0 | (0.0) |  |  |  |  | |  |
| Negative alcohol-related consequences |  | |  |  |  |  |  |  | |  | |
| Short Inventory of Problems (SIP) - – past 3 months, mean (SD) | 1.2 | | (0.2) | 1 | n/a | 2.6 | (0.4) | 6.8 | | (0.3) | |
| Short Inventory of Problems (SIP) - - Lifetime, mean (SD) | 4.5 | | (0.5) | 5 | n/a | 6.8 | (0.6) | 10.1 | | (0.3) | |
| Never seen anyone/gone anywhere for drinking-related reason? | 36 | | (73.5) | 0 | (0.0) | 18 | (56.3) | 81 | | (36.5) | |
| Somewhat or ready to change** | 22 | | (44.9) | 0 | (0.0) | 19 | (59.4) | 165 | | (74.3) | |
| Somewhat or very Important to change | 18 | | (36.7) | 0 | (0.0) | 21 | (65.6) | 178 | | (80.2) | |
| Somewhat or very confident in ability to change | 46 | | (93.9) | 1 | (100.0) | 28 | (87.5) | 171 | | (77.0) | |

* Sum of the total number of positive screens for depression, generalized anxiety, PTSD, panic, and DUD
