# Supplementary material for: Comparison of DSM-IV and DSM-5 criteria for alcohol use disorders in VA primary care patients with frequent heavy drinking enrolled in a trial
Source: Addict Sci Clin Pract. 2017 Jul 18;12:17. doi: 10.1186/s13722-017-0082-0 (PMC5514480; doi:10.1186/s13722-017-0082-0)
Supplement: Supplementary file 3 — Additional file 3. Individual Symptoms from the Short Inventory of Problems for Patients Meeting Criteria for Neither DSM-IV nor DSM-5 AUD, DSM-IV AUD alone, DSM-5 AUD alone, or Both. [file 13722_2017_82_MOESM3_ESM.docx]

Additional File 3: Individual Symptoms from the Short Inventory of Problems for Patients Meeting Criteria for Neither DSM-IV nor DSM-5 AUD, DSM IV AUD alone, DSM 5 AUD alone, or Both

|  | Neither DSM-IV nor DSM-5  n=42 | | DSM-IV  AUD alone  n=1 | | DSM-5  AUD alone  n=39 | | Both DSM-IV  & DSM-5 AUD  n=222 | |
| --- | --- | --- | --- | --- | --- | --- | --- | --- |
| Symptoms from the Short Inventory of Problems (SIP) in the past 3 months |  |  |  |  |  |  |  |  |
| 1. I have been unhappy because of my drinking | 5 | (11.9) | 0 | (0.0) | 14 | (35.9) | 150 | (67.6) |
| 2. Because of my drinking, I have not eaten properly | 6 | (14.3) | 0 | (0.0) | 13 | (33.3) | 150 | (67.6) |
| 3. I have failed to do what is expected of me because of my drinking | 3 | (7.1) | 0 | (0.0) | 7 | (17.9) | 111 | (50.0) |
| 4. I have felt guilty or ashamed because of my drinking | 3 | (7.1) | 0 | (0.0) | 11 | (28.2) | 129 | (58.1) |
| 5. I have taken foolish risks when I have been drinking | 5 | (11.9) | 1 | (100.0) | 5 | (12.8) | 108 | (48.6) |
| 6. When drinking, I have done impulsive things that I regretted later | 1 | (2.4) | 0 | (0.0) | 4 | (10.3) | 106 | (47.7) |
| 7. My physical health has been harmed by my drinking | 5 | (11.9) | 0 | (0.0) | 12 | (30.8) | 129 | (58.1) |
| 8. I have had money problems because of my drinking | 2 | (4.8) | 0 | (0.0) | 3 | (7.7) | 78 | (35.1) |
| 9. My physical appearance has been harmed by my drinking | 3 | (7.1) | 0 | (0.0) | 8 | (20.5) | 105 | (47.3) |
| 10. My family has been hurt by my drinking | 1 | (2.4) | 0 | (0.0) | 2 | (5.1) | 77 | (34.7) |
| 11. A friendship or close relationship has been damaged by my drinking | 1 | (2.4) | 0 | (0.0) | 3 | (7.7) | 61 | (27.5) |
| 12. My drinking has gotten in the way of my growth as a person | 3 | (7.1) | 0 | (0.0) | 8 | (20.5) | 110 | (49.5) |
| 13. My drinking has damaged my social life, popularity or reputation | 1 | (2.4) | 0 | (0.0) | 1 | (2.6) | 69 | (31.1) |
| 14. I have spent too much or lost a lot of money because of my drinking | 4 | (9.5) | 0 | (0.0) | 6 | (15.4) | 115 | (51.8) |
| 15. I have had an accident while drinking or intoxicated | 0 | (0.0) | 0 | (0.0) | 0 | (0.0) | 22 | (9.9) |
